# Genome-Wide Mining, Characterization, and Development of Microsatellite Markers in *Gossypium* Species

**DOI:** 10.1038/srep10638

**Published:** 2015-06-01

**Authors:** Qiong Wang, Lei Fang, Jiedan Chen, Yan Hu, Zhanfeng Si, Sen Wang, Lijing Chang, Wangzhen Guo, Tianzhen Zhang

**Affiliations:** 1 State Key Laboratory of Crop Genetics and Germplasm Enhancement, Cotton Hybrid R & D Engineering Center (the Ministry of Education), Nanjing Agricultural University, Nanjing 210095, China

## Abstract

Although much research has been conducted to characterize microsatellites and develop markers, the distribution of microsatellites remains ambiguous and the use of microsatellite markers in genomic studies and marker-assisted selection is limited. To identify microsatellites for cotton research, we mined 100,290, 83,160, and 56,937 microsatellites with frequencies of 41.2, 49.1, and 74.8 microsatellites per Mb in the recently sequenced *Gossypium* species: *G. hirsutum*, *G. arboreum,* and *G. raimondii,* respectively. The distributions of microsatellites in their genomes were non-random and were positively and negatively correlated with genes and transposable elements, respectively. Of the 77,996 developed microsatellite markers, 65,498 were physically anchored to the 26 chromosomes of *G. hirsutum* with an average marker density of 34 markers per Mb. We confirmed 67,880 (87%) universal and 7,705 (9.9%) new genic microsatellite markers. The polymorphism was estimated in above three species by *in silico* PCR and validated with 505 markers in *G. hirsutum*. We further predicted 8,825 polymorphic microsatellite markers within *G. hirsutum* acc. TM-1 and *G. barbadense* cv. Hai7124. In our study, genome-wide mining and characterization of microsatellites, and marker development were very useful for the saturation of the allotetraploid genetic linkage map, genome evolution studies and comparative genome mapping.

Microsatellites, which consist of a variable number of tandem repeats, are known as simple sequence repeats (SSRs, defined as 1-6 bp) and are characterized by their high frequency, distribution, co-dominance, reproducibility, and high polymorphism^1^,^2^. Among genetic marker systems such as restriction fragment length polymorphism (RFLP), random amplified polymorphic DNA (RAPD), amplified fragment length polymorphism (AFLP), sequence-related amplified polymorphism (SRAP), and target region amplification polymorphism (TRAP), microsatellites have numerous uses, including linkage map, quantitative trait loci (QTL) mapping, marker-assisted selection, genetic diversity studies, and evolutionary studies^3^,^4^,^5^,^6^.

Cotton (*Gossypium* SPP.) is the most important fiber crop in the word and is also an important edible oil crop. The genus *Gossypium* has nine genome types: eight diploid genomes (A, B, C, D, E, F, G, and K; 2*n* = 2*x* = 26) and one tetraploid genome (AD; 2*n *= 4*x* = 52), based on observations of chromosome pairing^7^,^8^,^9^. Two of the four cultivated allotetraploid species, *G. hirsutum* L. (AD)_1_ and *G. barbadense* L. (AD)_2_, account for 90% and 8% of world cotton production, respectively^5^. Although *G. arboreum* (A_2_) and *G. raimondii* (D_5_) are regarded as the progenitors that led to the formation of the tetraploid cotton species 1-2 million years ago (MYA), the exact donor species that no longer exist^10^.

Much research has been conducted to characterize microsatellites and develop markers from enriched genomic DNA^11^,^12^, expressed sequence tags^13^,^14^,^15^, sequences derived from bacterial artificial chromosomes^16^,^17^, assembled contigs based on transcriptomic profile^18^ and sequenced genome^19^. Current publicly available cotton simple sequence repeat (SSR) markers are described in the CottonDB (http://cottondb.org/) and Cotton Marker Database (CMD) ( http://cottonmarker.org)^20^. So far, 19,010 SSR markers have been described in the CottonDB. CottonGen supersedes CottonDB and the CMD with enhanced tools for easier data sharing, mining, visualization and data retrieval of cotton research data^21^. Thanks to global efforts, high-resolution mapping in cotton has been published with segregating populations through interspecific crosses^19^,^22^,^23^,^24^,^25^. However, the development of microsatellites in cotton remains limited, due to the paucity of DNA polymorphisms and the lack of complete genome sequences^26^,^27^,^28^. Although a very-high-density whole genome marker map (WGMM) has been constructed for cotton based on the D genome, which has a total of 48,959 loci, about six times the number characterized in the most richly populated of the integrated maps published previously^20^,^29^, the pattern of microsatellite distribution and the physical position or product of publicly available cotton SSRs in *G. hirsutum* remained ambiguous.

Recent developments in high throughput DNA sequencing technologies provide new opportunities to expedite molecular marker development^30^. In this study, we conducted whole-genome microsatellite characterization and marker development in the sequenced genome of *G. hirsutum*^31^, *G. raimondii*^32^, and *G. arboreum*^33^. We characterized and compared the frequency and distribution of motif length, type, and repeat number of microsatellites in the assembled genomic sequences of these three species. In addition, we analyzed the genomic distribution of microsatellites, genes and transposable elements (TEs) in the assembled genome of *G. hirsutum*. Furthermore, we developed microsatellite markers from assembled genomic sequences, and evaluated polymorphism in different species. These markers may facilitate the advancement of many basic and applied genomic studies in cotton, including the development of high-resolution linkage maps, positional gene-cloning, and breeding in different cotton species.

## Results and discussion

### Frequency, distribution and characterization of microsatellite length, type and number

The available 2,433 Mb, 1,694 Mb and 761 Mb genome sequences of *G. hirsutum*, *G. arboreum,* and *G. raimondii,* respectively, were searched for microsatellites with different types of desirable repeat motifs from mono- to hexanucleotide. A total of 100,290, 83,160, and 56,937 microsatellites were identified with an overall frequency of 41.2, 49.1, and 74.8 per Mb, or one every 24.3, 20.4 and 13.4 Kb, in above three assembled genomic sequences, respectively (Table 1). Meanwhile, a total of 50,443 and 41,119 microsatellites with perfect repeats were detected in the A_t_ and D_t_, respectively. These showed an overall density of 34.2 (A_t_, “t” indicates tetraploid) and 49.5 (D_t_) per Mb, or one every 29.3 and 20.2 Kb, respectively (Table 1). The determination of the genomic distribution of 100,290 microsatellites revealed 81,898 microsatellites mapped to the 26 chromosomes of *G. hirsutum* with an average density of 42.33 per Mb. In our study, physically mapped microsatellites showed a higher density of markers on the D_t_ (38,622, 49.87/Mb) than the A_t_ (43,276, 37.30/Mb), with maximums on A05 (4,248, 46.15/Mb) and D05 (3,553, 57.37/Mb), and minimums on A06 (3,204, 31.06/Mb) and D02 (3091, 45.94/Mb) (Table S1). Our results agree with a previous study which revealed a negative correlation between genome size and microsatellite density^34^. Cucumber (367 Mb), wheat (3B, 1,000Mb), and maize (1,115 Mb) have microsatellite densities of 551.9 per Mb, 163 per Mb and 120 per Mb, respectively^4^,^35^,^36^. Other microsatellite densities of 78.5 per Mb, 189.4 per Mb and 99.8 per Mb have been identified in *Brachypodium*, rice, and sorghum^37^.

**Figure 1:**
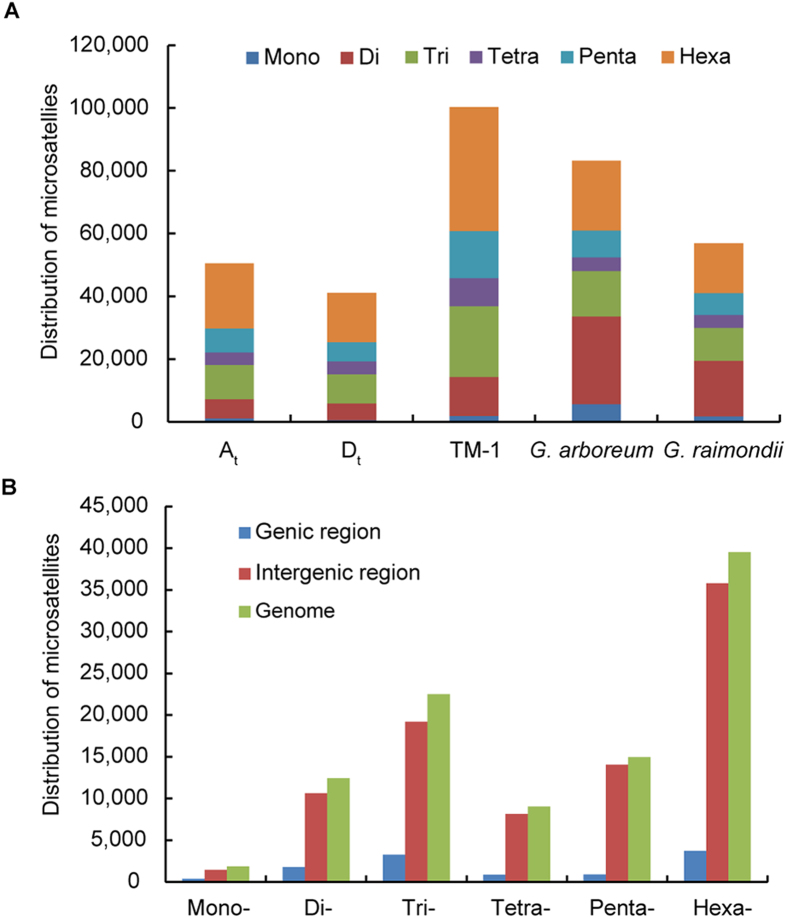
Distribution of microsatellites identified in the assembled genomic sequences of *G. hirsutum* (TM-1), *G. arboreum* and *G. raimondii*. (**A**) Frequency distribution of microsatellites with different motif lengths in *Gossypium* species. (**B**) Distribution of microsatellites in genic and intergenic regions in *G. hirsutum* (TM-1).

The distributions of microsatellite length in the assembled genomic sequences of *G. hirsutum*, *G. arboreum* and *G. raimondii* showed that tri-, tetra-, penta- and hexanucleotide repeats accounted for very similar proportions, whereas mono- and dinucleotide repeats were relatively different in proportions among these genomes (Fig.1A). Among the 100,290 microsatellites obtained in *G. hirsutum*, the hexanucleotide repeats were most abundant (39,506) with a proportion of 39.4%, followed by tri- (22,483, 22.4%), penta- (14970, 14.9%), di- (12,445, 12.4%), tetra- (9,031, 9.0%), and mono-nucleotide (1,855, 1.8%) (Fig.1A, Table S2). This distribution pattern of microsatellite length differed to earlier reports that di-nucleotide repeats (DNR) are abundant in rice and *Arabidopsis*^38^, and tri-nucleotide repeats (TNR) are abundant in *Brachypodium*^37^, *bamboo*^39^, switchgrass^40^, and Foxtail Millet^41^. In cotton, penta-nucleotide repeats (PNR) were found to be most abundant in the *G. raimondii* genome^42^. Some of these differences may be due to variations in characterization parameters, algorithms and bioinformatics software^43^,^44^. In principle, a microsatellite can extend to any length in the absence of selection force^38^. Generally, shorter repeat lengths were used to define microsatellite in *Brassica*^45^, Foxtail Millet^41^, and *G. raimondii*^42^. The relax criteria was also used to identify microsatellite with minimum repeat lengths of 12, 6, 4, 3, 3, and 3; showing that the proportion of mono- to hexanucleotide repeats is similar in *G. hirsutum*, *G. arboreum* and *G. raimondii* (Fig. S1). The different results of these two criteria (minimum microsatellite length 18 vs 12) mostly due to longer reads used in genome assembling of *G. raimondii* such as Roche 454 data^32^. Among these 100,290 microsatellites in *G. hirsutum*, 11,008 (11.0%) were detected in the genic regions. More intergenic microsatellites from mono- to hexanucleotide were identified than that in genic regions (Fig.1B).

The distributions of microsatellite type in the assembled genomic sequences of *G. hirsutum*, *G. arboreum,* and *G. raimondii* are presented in Fig. S2A and Table S2. Specifically, the major motifs were rich in A, AT, AAT/AAG, AAAT, AAAAT and AAAAAT, and the minor motifs were mostly riched in C/G (Fig. S2A, Table S2 and S3), which is consistent with previous reports on microsatellites from the assembled genomic sequences of *G. raimondii*^42^, *Cucumis sativus*^4^ and *Brassica napus*^45^. It was noted that the nucleotide composition characteristics of these A/T and C/G motifs corresponded well with their higher A/T than C/G content, such as 34.1% GC content in *G. hirsutum*^31^. However, AAT was the most common motif in allotetraploid *G. hirsutum*, whereas AT was most common in diploid *G. arboreum* and *G. raimondii*. More interestingly, we found significantly higher levels of AATCAG in A_t_ (2,590) and *G. arboreum* (2,646) compared to D_t_ (42) and *G. raimondii* (43), but much lower number of AACCCT motif were found in A_t_ (60) and *G. arboreum* (73) compared to D_t_ (332) and *G. raimondii* (421). In addition, the ACAGG repeat was only detected in A_t_ (283) and *G. arboreum* (224) (Table S2).

**Figure 2:**
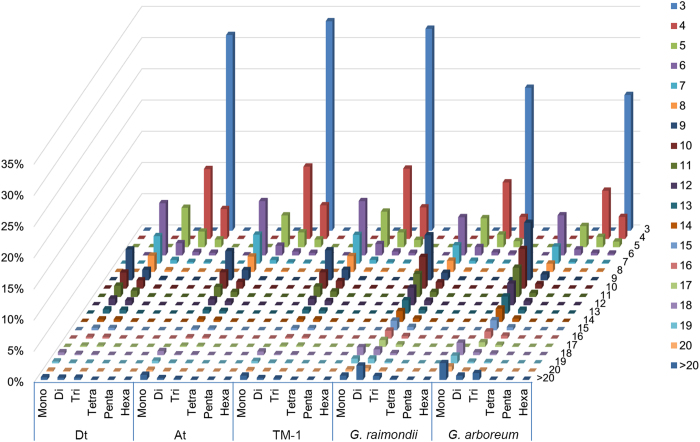
Distribution of SSR motif repeat numbers from mono- to hexanucleotide.The vertical axis shows the abundance of microsatellites that have different motif repeat numbers (from 3 to >20), which are discriminated by legends of different colors.

The distributions of microsatellite repeat numbers in the assembled genomic sequences of *G. hirsutum*, *G. arboreum* and *G. raimondii* revealed that microsatellite frequency decreased as the number of repeat units increased (Fig. S2B). Interestingly, as the motif repeat number increased, the microsatellite abundances decreased and the rate of change was slowest for dinucleotides, followed by trinucleotide repeats (Fig. 2). These finding are similar to the research on *Brassica*^45^. Although we found that the number of mono- and dinucleotide was dramatically reduced in *G. hirsutum*, high correlations were identified between these genomic sequences based on motif repeat numbers (Table S4).

In conclusion, almost all of the analyzed characteristics of microsatellite distribution in assembled genome of *G. hirsutum* and its two progenitors were highly similar, which suggests that the pattern of microsatellite distribution is conservative in *Gossypium* or retained after formation of allotetraploid cotton. This is understandable because allotetraploid cotton is reunited by *G. arboreum* and *G. raimondii* approximately 1-2 MYA ago^12^.

### Genomic distribution

**Figure 3:**
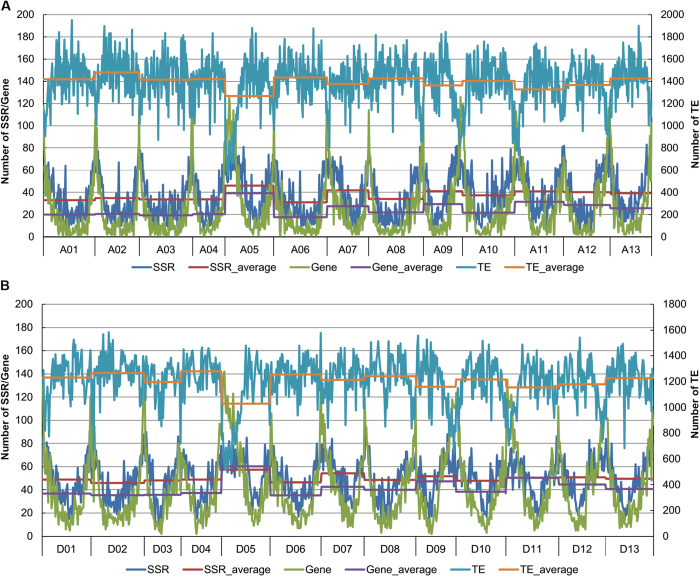
The genomic distributions of microsatellites, genes, and TEs in the assembled genome of *G. hirsutum*. (**A**) Genome-wide distributions of microsatellites, genes, and TEs in the A_t_. (**B**) Genome-wide distributions of microsatellites, genes, and TEs in the D_t_. The horizontal axis indicates the chromosomes A01-A13 and D01-D13, and each chromosome was divided into 1-Mb for statistical analysis of microsatellites, genes, and TEs. The left vertical axis indicates the frequencies of the microsatellites and genes, and the right vertical axis indicates the frequencies of TE. The curves represent practical frequencies and the lines show average frequencies.

Based on the assembled genome of *G. hirsutum*, the genomic distributions of microsatellites, genes and transposable elements were investigated. We illustrated greater physical densities in distal chromosomal regions than in the central regions; in agreement with a previous study that found a greater marker density in the distal gene-rich ends of the chromosomes^20^. Specifically, the genomic distribution of microsatellites was positively correlated with genes and negatively correlated with TEs (Fig. 3, Table S5). For both A_t_ and D_t_, the frequencies of microsatellites in the 1-Mb genomic intervals were significantly positively correlated with genes (r = 0.78 and 0.73) and negatively correlated with TEs (r = −0.34 and −0.14). These results are similar to previous reports that microsatellites are associated with gene sequences in plants^34^,^45^. It was interesting that the homoeologous chromosomes A05 and D05 exhibited the highest average frequency of microsatellites on the A_t_ and D_t_, respectively.

### Development of genome-wide microsatellite markers

A total of 77,996 (83.2%), 63,263 (81.46%) and 44,388 (82.8%) identified microsatellite markers were designed from the flanking sequences of 93,736, 77,661, and 53,586 microsatellites from the genomic sequences of *G. hirsutum*, *G. arboreum* and *G. raimondii*, respectively. All of the 77,996 developed microsatellite markers were summarized with motif length, amplification length and polymorphism in Table S6. The remaining microsatellite markers failed to generate specific amplicons mostly due to the limited number of flanking sequences from each side of identified microsatelltes. Similar observations have been reported in the mining of genome microsatellites in other crop plants^37^,^41^,^46^.

The most useful microsatellites are those where the chromosome location is known on the assembled chromosomes. The physical location of 65,498 of the 77,996 microsatellite markers was revealed on the 26 chromosomes of *G. hirsutum,* with average marker density of 33.86 markers per Mb. The average marker density of 40.64 markers/Mb on the D_t_ was larger than the 29.33 markers/Mb on A_t_. The maximum density (47.07 markers/Mb) was found on D05, followed by 44.35 markers/Mb on D07, and 42.27 markers/Mb on D09, and the minimum density (24.81 markers/Mb) were found on A06 (Table 2). The physical positions of the developed markers across the 26 chromosomes can provide a high-density microsatellite map that contributes to genome-wide MAS in research of gene mapping, and to comparative genome mapping involving *G. hirsutum* and related crop plants. Only 10,116 (13%) identified microsatellite markers were available in the previous public database (www.cottongen.org), and the remaining 67,880 (87%) were universal.

Although several studies have been conducted to develop genic microsatellite markers from Expressed Sequence Tag (ESTs) transcripts of *G. arboreum*^14^, *G. raimondii*^47^,^48^, and *G. hirsutum*^48^, the number of publicly available genic microsatellite markers in *Gossypium* was limited (http://www.cottongen.org). We have identified 10,449 (13.4%) genic microsatellite markers known as “functional markers” that have a high transferability across species. Of them, 7,705 were new genic markers by crosschecking within CottonGen and relative literatures. There were more intergenic microsatellites markers than genic markers as a result of 8.64% genic region in *G. hirsutum* genome, although microsatellites are preferentially associated with nonrepetitive DNA in plant genomes^34^. Microsatellites in coding regions can regulate gene expression or function, and the mutation rate in coding sequences is lower than in noncoding sequences, therefore, the number of SSRs and polymorphisms is lower in coding regions^49^. The main advantage of developing genic microsatellite markers is the possibility of finding associations between functional genes and phenotypes^50^,^51^.

### *In silico* PCR analysis

To avoid complicated errors in genotyping due to the polyploidy nature of *G. hirsutum*, all the developed genome-wide microsatellite markers were subjected to *in silico* PCR analysis based on the genome sequences of *G. hirsutum*, *G. raimondii* and *G. arboreum* (Table 3). As to 77,996 microsatellite markers designed in *G. hirtusum*, 0 (0.0%), 29,392 (37.7%), 20,911 (26.8%), 5,151 (6.6%), and 22,542 (28.9%) markers generated 0, 1, 2, 3, > 3 *in silico* PCR products from the *de novo* sequences of *G. hirsutum*, respectively (Table 3 and Table S6). Among all these microsatellite markers, an average of forty eight *in silico* PCR products were identified, because some element (TE) associated markers could generate tens of thousands of *in silico* products, as reported in *Brassica*^45^. We also found 62,326 (79.9%) markers generating ≤10 *in silico* PCR products with an average of two alleles, and four alleles for 68,811 (88.2%) markers generating ≤50 *in silico* PCR products (Fig. S3).

Through *in silico* PCR analysis, a set of 20,911 (26.8%) high-quality double-locus microsatellite markers and 29,392 (37.7%) single-locus were established. In diploid species such as barley and rice, most microsatellite markers are single locus amplifying a maximum of two alleles. However, in allotetraploid *G. hirsutum*, most microsatellite markers are found in multiple loci and amplify multiple alleles from homoeologous loci. This limits their application in genetic and breeding studies^52^. Microsatellite markers that generate one *in silico* PCR product are likely to be more useful, as is the case of single locus SSR markers developed by practical PCR amplification in inbred lines^53^ and genome-wide development^45^.

Furthermore, as to 77,996 microsatellite markers designed in *G. hirtusum*, 32,868 (42.1%), 30,588 (39.2%), 2,876 (3.7%), 1,391 (1.8%), and 10,273 (13.2%) markers generated 0, 1, 2, 3 and > 3 *in silico* PCR product from the genomic sequences of *G. raimondii*, respectively and 25,026 (32.1%), 33,406 (42.8%), 3,388 (4.3%), 1,501 (1.9%), and 14,675 (18.8%) markers generated 0, 1, 2, 3, and>3 *in silico* PCR product from the genomic sequences of *G. arboreum*, respectively.

### Application and experimental evaluation of microsatellite markers

A total of 511 from 77,996 microsatellite markers were selected to evaluate the amplification (Table S7). In our result, 505 (98.8%) of the 511 microsatellite markers could produce clear and reproducible amplification products with 996 polymorphic alleles (Table S7). Among these 505 microsatellite markers, 266 (52.7%) amplified the corresponding polymorphic alleles with *in silico* PCR products. Furthermore, 131 (29.4%) amplified more polymorphic alleles than *in silico* PCR products, which may be caused by the high proportion of TEs in the *Gossypium* genome. Therefore, these genomic microsatellite markers would be of enormous of use for various genotyping applications.

To generate microsatellite markers with the potential to enhance the genetic map, we tested the polymorphisms of 77,996 developed microsatellite markers in TM-1 and Hai7124 using re-sequencing data of Hai7124 (111.8 GB, 51,526 SSRs). Approximately 9,001 (11.5%) of these microsatellite markers were polymorphic. Of the 9,001 microsatellite markers, 8,825 were mapped to 26 chromosomes, including 4,446 on the A_t_ and 4,379 on the D_t_. The largest proportions were found on A12 and D02 (Table S8). Thirty microsatellite markers were randomly selected to amplify polymorphisms between TM-1 and Hai7124, successfully validating the accuracy of developed markers (Fig. S4). The first comprehensive SSR maps were reported in studies using 138 BC_1_ plants derived from an interspecific cross of (*G. hirsutum* acc.TM-1 × *G. barbadense* cv. Hai 7124) × TM-1^48^. In order to construct a high-density genetic map of cotton in our laboratory, we have tried EST-SSRs, SNPs, and InDels when no sufficient markers in cotton were available^54^. Thus, these 8,825 polymorphic microsatellite markers are a useful resource for enhancing the genetic map and improving molecular marker assisted selection breeding.

In the present study, we conducted a genome-wide analysis to identify 100,290 microsatellites in *G. hirsutum* and developed 77,996 microsatellite markers. Among these markers, 67,880 (87%) were universal and 7,705 were new genic microsatellite markers. These genome-wide microsatellite markers were useful in genotyping applications such as germplasm characterization and high-density microsatellite marker linkage map construction. Importantly, the physical positions of the universal microsatellite markers on 26 chromosomes and identification of polymorphic marker can provide a high-density microsatellite map that contributes to genome-wide microsatellite marker selection in research areas such as gene fine mapping, MAS breeding, and comparative genome mapping involving *G. hirsutum* and related crop plants.

### Experimental procedures

#### Sources of genomic sequences

The high-quality genome sequence of the genetic standard line of Upland cotton, TM-1 (PRJNA248163), is available in http://mascotton.njau.edu.cn. The genomic sequence of G. *raimondii*^32^ was downloaded from http://phytozome.net and the genomic sequence of G. *arboreum*^33^ was downloaded from http://cgp.genomics.org.cn/page/species/index.jsp.

#### Identification of microsatellites

Genome sequences were searched for perfect microsatellites using PERL5 script MIcroSAtellite (MISA, http://pgrc.ipk-gatersleben.de/misa/) with basic motifs from mono- to hexanucleotide^55^. Repeats with a minimum of 18, 9, 6, 5, 4, and 3 were defined for the mono- to hexanucleotide, respectively. Compound microsatellites were defined as ≥2 repeats interrupted by ≤100 bp, as previous report^48^.

#### Statistical analysis

Each chromosome was divided into 1-Mb for statistical analysis of microsatellites, genes, and TEs for the represent practical frequencies and average frequencies. Then, Excel statistical function CHISQ.TEST was used to calculate the significance level (*P*_*x^2^* test_) of these two frequencies of microsatellites as well as genes and TEs in 26 chromosomes.

#### Design of SSR primers

Primer pairs were designed from the flanking sequences of identified microsatellites using PRIMER3 software^56^, and two perl scripts, p3_in.pl and p3_out.pl served as interface modules between MISA and Primer3 with the primer designing parameters: 18–27 bp in length, 57–63 °C in melting temperature, 30–70% in GC content and 100–280 bp in product size. These two perl scripts were downloaded from MISA ( http://pgrc.ipk-gatersleben.de/misa/). Primer3 was downloaded from http://www-genome.wi.mit.edu/genome_software/other/primer3.html. The p3_in.pl was used to create a primer3 input file which was submitted to Primer3. Then p3_out.pl was used to calculated and merge all information together.

#### *In silico* analysis of microsatellite polymorphisms

The primer-pair sequences of previously developed publicly available *Gossypium* SSR markers were downloaded from the CottonGen website (www.cottongen.org). In our study, all microsatellite markers were aligned to genomic sequences of *G. hirsutum*, *G. arboreum* and *G. raimondii* using an *in silico* PCR strategy with the following default parameters: 2 bp mismatch, 1 bp gap, 50 bp margin and 50–1000 bp product size, as previous report^45^,^57^. The software (e-PCR-2.3.11) used for *in silico* PCR was downloaded from ftp://ftp.ncbi.nlm.nih.gov/pub/schuler/e-PCR/. And only one genome was used at a time.

#### Evaluation of polymorphisms in *G. hirsutum* and *G. barbadense*

Illumina reads of *G. barbadense* cv. Hai7124 (Biosample: SAMN03002317) were mapped to the genome of *G. hirsutum* acc. TM-1 using the Burrows-Wheeler Alignment tool (BWA ver 0.6.2)^58^, with mainly default parameters. SAMtools^59^ was used to call InDels ≥5 with mapped reads of a minimum mapping quality of 20. If InDels were located in the region of paired primers/microsatellite markers, we regarded the paired primer as a putative polymorphic primer between TM-1 and Hai7124.

### Data Section

The detail information of 77,996 developed markers and *in silico* PCR amplification were deposited in Table S6. These markers will be publically available in http://mascotton.njau.edu.cn and CottonGen database.

## Additional Information

**How to cite this article**: Wang, Q. *et al.* Genome-Wide Mining, Characterization, and Development of Microsatellite Markers in *Gossypium* Species. *Sci. Rep.* **5**, 10638; doi: 10.1038/srep10638 (2015).

## Supplementary Material

Supporting Information

Supporting Information

Supporting Information

Supporting Information

## Figures and Tables

**Table 1:** Overall frequency of microsatellites in *Gossypium* species.

**Species**	**Microsatellites number**	**Genome length (Mb)**	**Frequency**	
			**Per Mb**	**One every (Kb)**
A_t_	50,443	1477.1	34.2	29.3
D_t_	41,119	831.0	49.5	20.2
*G. hirsutum*	100,290	2432.7	41.2	24.3
*G. arboreum*	83,160	1694.6	49.1	20.4
*G. raimondii*	56,937	761.4	74.8	13.4

A_t_, D_t_: two subgenomes of allotetraploid cotton *G. hirsutum.*

**Table 2:** Summary of chromosomal distribution and average density of microsatellite markers mapped on *G. hirsutum* chromosomes.

**Chr.**	**Marker mapped**	**Density (Per Mb)**	**Chr.**	**Marker mapped**	**Density(Per Mb)**
A01	2612	26.15	D01	2452	39.90
A02	2345	28.10	D02	2491	37.02
A03	2592	25.85	D03	1849	39.60
A04	1682	26.74	D04	2038	39.61
A05	3373	36.64	D05	2915	47.07
A06	2560	24.81	D06	2430	37.79
A07	2571	32.86	D07	2453	44.35
A08	2794	26.96	D08	2606	39.55
A09	2405	32.07	D09	2166	42.47
A10	2884	28.59	D10	2438	38.47
A11	3028	32.45	D11	2712	41.04
A12	2732	31.23	D12	2493	42.18
A13	2447	30.60	D13	2430	40.14
**A**_**t**_	**34025**	**29.33**	**D**_**t**_	**31473**	**40.64**
**All**	**65498**	**33.86**			

Chr.: Chromosome A_t_, D_t_: two subgenomes of allotetraploid cotton *G. hirsutum.*

**Table 3:** Generated number (%) of *in silico* PCR products by genome-wide microsatellite markers in the sequenced genome of *G. hirsutum*, *G. raimondii* and *G. arboreum*.

**Markers from**	***in silico*** **PCR in**	**Zero**	**One**	**Two**	**Three**	** > Three**	**Total**
	*G. raimondii*	0(0.0)	30920(69.7)	2912(6.6)	1441(3.2)	9115(20.5)	
*G. raimondii*	*G. arboreum*	26647(60.0)	12706(28.6)	1326(3.0)	523(1.2)	3186(7.2)	44388 (100)
	*G. hirsutum*	6532(14.7)	14134(31.8)	11553(26.0)	2345(5.3)	9824(22.1)	
							
	*G. raimondii*	42616(67.4)	14973(23.7)	1238(2.0)	545(0.9)	3891(6.2)	
*G. arboreum*	*G. arboreum*	0(0.0)	37060(58.6)	4543(7.2)	1761(2.8)	19899(31.5)	63263 (100)
	*G. hirsutum*	6694(10.6)	19880(31.4)	14138(22.3)	3061(4.8)	19490(30.8)	
							
	*G. raimondii*	32868(42.1)	30588(39.2)	2876(3.7)	1391(1.8)	10273(13.2)	
*G. hirsutum*	*G. arboreum*	25026(32.1)	33406(42.8)	3388(4.3)	1501(1.9)	14675(18.8)	77996 (100)
	*G. hirsutum*	0(0.0)	29392(37.7)	20911(26.8)	5151(6.6)	22542(28.9)	
