# Supplementary material for: Genome-Wide Mining, Characterization, and Development of Microsatellite Markers in Gossypium Species
Source: Sci Rep. 2015 Jun 1;5:10638. doi: 10.1038/srep10638 (PMC4650602; doi:10.1038/srep10638)
Supplement: Supporting Information [file srep10638-s1.doc]

**Genome-Wide Mining, Characterization, and Development of Microsatellite Markers in *Gossypium* Species**

Qiong Wang*, Lei Fang*, Jiedan Chen, Yan Hu, Zhanfeng Si, Sen Wang, Lijing Chang, Wangzhen Guo, Tianzhen Zhang†

State Key Laboratory of Crop Genetics and Germplasm Enhancement, Cotton Hybrid R & D Engineering Center (the Ministry of Education), Nanjing Agricultural University, Nanjing 210095, China.

^*^These authors contributed equally to this work.

†Correspondence and requests for materials should be addressed to TZ (cotton@njau.edu.cn).

**Supplementary Figures**

**
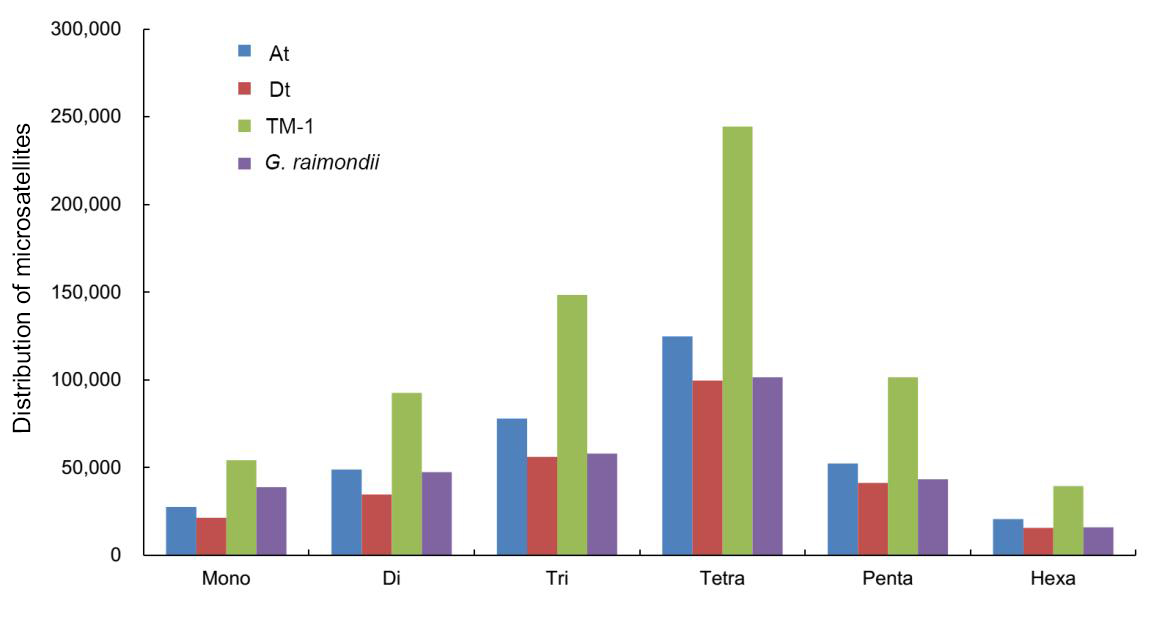
**

**Figure S1.** Distribution of microsatellite motif length from mono- to hexanucleotide repeats in *G. hirsutum* and *G. raimondii*. A_t_, D_t_: two subgenomes of allotetraploid cotton *G. hirsutum.* Flexible relaxed criteria were used to identify microsatellites with minimum repeat lengths of 12, 6, 4, 3, 3, and 3*.*

**
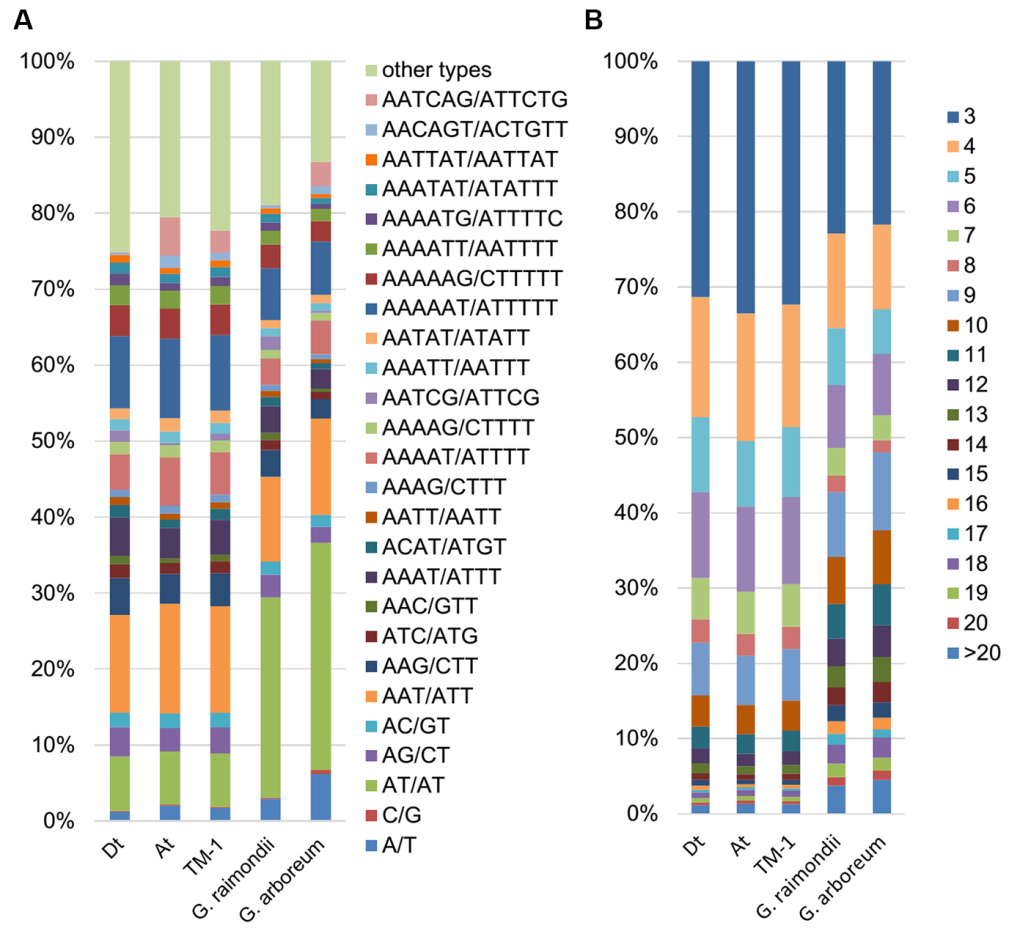
**

**Figure S2.** Distribution of microsatellite motif type (A) and repeat number (B) in *Gossypium* species. The vertical axis indicats the abundance (%) of microsatellites with different motif lengths, types or repeat numbers that are discriminated in the legends using different colors.


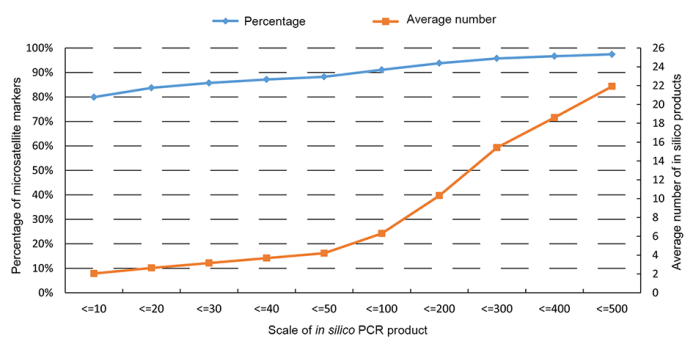


**Figure S3.** Average number of *in silico* PCR products based on different sacles. Blue line indicated the percentage of microsatellite markers with different allele ranges according to *in silico* PCR product. Orange line indicated the average number of *in silico* PCR in different allele ranges.


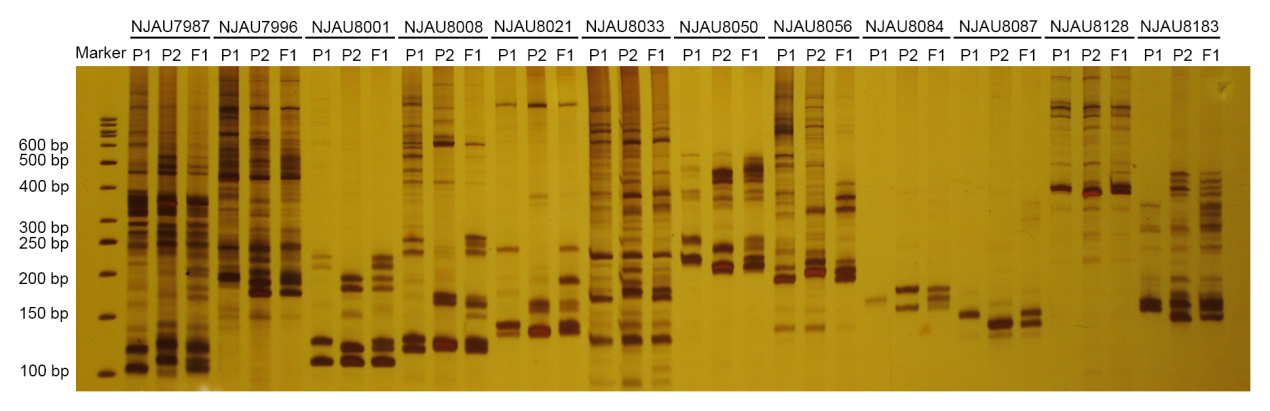


**Figure S4.** Amplification of microsatellite markers between *G. hirsutum* and *G. barbadense*. Genotyping results were found in P1 (*G. hirsutum* TM-1), P2 (*G. barbadense* Hai7124) and F_1_ (TM-1 × Hai 7124).

**Supplementary Tables**

**Table S1:** Summary of chromosomal distribution and average density of microsatellites mapped on *G. hirsutum* chromosomes.

| **Chr.** | **Mono-** | **Di-** | **Tri-** | **Tetra-** | **Penta-** | **Hexa-** | **Marker mapped** | **Density marker ( Per MB)** | **Chr.** | **Mono-** | **Di-** | **Tri-** | **Tetra-** | **Penta-** | **Hexa-** | **Marker mapped** | **Density marker (Per MB)** |
| --- | --- | --- | --- | --- | --- | --- | --- | --- | --- | --- | --- | --- | --- | --- | --- | --- | --- |
|
| A01 | 77 | 419 | 705 | 240 | 495 | 1367 | 3303 | 33.07 | D01 | 39 | 397 | 732 | 296 | 443 | 1097 | 3004 | 48.88 |
| A02 | 61 | 338 | 644 | 229 | 454 | 1181 | 2907 | 34.84 | D02 | 40 | 409 | 656 | 329 | 477 | 1180 | 3091 | 45.94 |
| A03 | 70 | 405 | 668 | 291 | 530 | 1405 | 3369 | 33.60 | D03 | 33 | 260 | 467 | 241 | 346 | 904 | 2251 | 48.21 |
| A04 | 38 | 250 | 473 | 176 | 323 | 863 | 2123 | 33.74 | D04 | 36 | 333 | 567 | 238 | 381 | 958 | 2513 | 48.84 |
| A05 | 110 | 539 | 973 | 349 | 634 | 1643 | 4248 | 46.15 | D05 | 56 | 540 | 854 | 328 | 486 | 1289 | 3553 | 57.37 |
| A06 | 61 | 382 | 661 | 254 | 554 | 1292 | 3204 | 31.06 | D06 | 43 | 350 | 604 | 300 | 511 | 1181 | 2989 | 46.49 |
| A07 | 46 | 409 | 761 | 245 | 499 | 1308 | 3268 | 41.76 | D07 | 29 | 362 | 741 | 290 | 438 | 1146 | 3006 | 54.35 |
| A08 | 70 | 465 | 767 | 272 | 550 | 1403 | 3527 | 34.04 | D08 | 37 | 434 | 705 | 343 | 498 | 1187 | 3204 | 48.62 |
| A09 | 59 | 352 | 708 | 249 | 481 | 1228 | 3077 | 41.03 | D09 | 37 | 354 | 606 | 229 | 380 | 1033 | 2639 | 51.75 |
| A10 | 78 | 427 | 882 | 294 | 571 | 1512 | 3764 | 37.32 | D10 | 35 | 391 | 662 | 320 | 460 | 1163 | 3031 | 47.83 |
| A11 | 82 | 489 | 927 | 329 | 540 | 1458 | 3825 | 40.99 | D11 | 51 | 400 | 774 | 326 | 481 | 1306 | 3338 | 50.51 |
| A12 | 78 | 427 | 837 | 280 | 520 | 1381 | 3523 | 40.27 | D12 | 30 | 399 | 678 | 308 | 437 | 1149 | 3001 | 50.77 |
| A13 | 65 | 410 | 714 | 244 | 433 | 1272 | 3138 | 39.24 | D13 | 28 | 358 | 721 | 293 | 460 | 1142 | 3002 | 49.59 |
| **At** |  |  |  |  |  |  | **43276** | **37.30** | **Dt** |  |  |  |  |  |  | **38622** | **49.87** |
| **All** |  |  |  |  |  |  | **81898** | **42.33** |  |  |  |  |  |  |  |  |  |

A_t_, D_t_: two subgenomes of allotetraploid cotton *G. hirsutum.*

**­**

**Table S2: Integrated analysis of the mono- to hexanucleotide repeats of microsatellites.**

| **Motif** | **D_t_** | | |  | **A_t_** | | |  | ***G. hirsutum* (TM-1)** | | |  | ***G. raimondii*** | | |  | ***G. arboreum*** | | |
| --- | --- | --- | --- | --- | --- | --- | --- | --- | --- | --- | --- | --- | --- | --- | --- | --- | --- | --- | --- |
|  | **Number (%)** | | **Repeat number** |  | **Number (%)** | | **Repeat number** |  | **Number (%)** | | **Repeat number** |  | **Number (%)** | | **Repeat number** |  | **Number (%)** | | **Repeat number** |
| Mono | **547** | 1.3 |  |  | **1103** | 2.2 |  |  | **1855** | 1.8 |  |  | **1754** | 3.1 |  |  | **5595** | 6.7 |  |
| A | 494 | 1.2 | 176 |  | 1028 | 2 | 366 |  | 1722 | 1.7 | 366 |  | 1649 | 2.9 | 39 |  | 5143 | 6.2 | 2203 |
| C | 53 | 0.1 | 25 |  | 75 | 0.1 | 29 |  | 133 | 0.1 | 8779 |  | 105 | 0.2 | 27 |  | 452 | 0.5 | 40 |
| Di | **5315** | 12.9 |  |  | **6034** | 12 |  |  | **12445** | 12.4 |  |  | **17691** | 31.1 |  |  | **27913** | 33.6 |  |
| AT | 2954 | 7.2 | 21 |  | 3529 | 7 | 217 |  | 7079 | 7.1 | 217 |  | 15010 | 26.4 | 177 |  | 24864 | 29.9 | 350 |
| AG | 1579 | 3.8 | 198 |  | 1529 | 3 | 109 |  | 3448 | 3.4 | 198 |  | 1693 | 3 | 69 |  | 1752 | 2.1 | 153 |
| AC | 779 | 1.9 | 328 |  | 976 | 1.9 | 286 |  | 1915 | 1.9 | 328 |  | 986 | 1.7 | 61 |  | 1297 | 1.6 | 6988 |
| CG | 3 | 0 | 9 |  | 0 | 0 | - |  | 3 | 0 | 9 |  | 2 | 0 | 9 |  | 0 | 0.0 | - |
| Tri | **9302** | 22.6 |  |  | **10987** | 21.8 |  |  | **22483** | 22.4 |  |  | **10475** | 18.4 |  |  | **14583** | 17.5 |  |
| AAT | 5290 | 12.9 | 86 |  | 7289 | 14.5 | 165 |  | 14077 | 14 | 165 |  | 6350 | 11.2 | 186 |  | 10553 | 12.7 | 5413 |
| AAG | 2000 | 4.9 | 118 |  | 1974 | 3.9 | 149 |  | 4308 | 4.3 | 149 |  | 1999 | 3.5 | 34 |  | 2111 | 2.5 | 1017 |
| ATC | 757 | 1.8 | 23 |  | 730 | 1.4 | 26 |  | 1634 | 1.6 | 26 |  | 757 | 1.3 | 30 |  | 811 | 1.0 | 185 |
| AAC | 464 | 1.1 | 29 |  | 308 | 0.6 | 22 |  | 853 | 0.9 | 29 |  | 539 | 0.9 | 31 |  | 351 | 0.4 | 26 |
| AGG | 235 | 0.6 | 12 |  | 243 | 0.4 | 14 |  | 459 | 0.5 | 14 |  | 226 | 0.4 | 27 |  | 211 | 0.3 | 16 |
| ACC | 227 | 0.6 | 14 |  | 191 | 0.5 | 14 |  | 524 | 0.5 | 14 |  | 242 | 0.4 | 18 |  | 269 | 0.3 | 17 |
| AGC | 152 | 0.4 | 12 |  | 124 | 0.2 | 19 |  | 297 | 0.3 | 19 |  | 153 | 0.3 | 13 |  | 135 | 0.2 | 13 |
| ACT | 108 | 0.3 | 32 |  | 68 | 0.1 | 29 |  | 187 | 0.2 | 32 |  | 135 | 0.2 | 25 |  | 79 | 0.1 | 68 |
| CCG | 36 | 0.1 | 11 |  | 37 | 0.1 | 9 |  | 79 | 0.1 | 11 |  | 39 | 0.1 | 11 |  | 40 | 0.0 | 12 |
| ACG | 33 | 0.1 | 8 |  | 23 | 0 | 16 |  | 65 | 0.1 | 16 |  | 35 | 0.1 | 9 |  | 23 | 0.0 | 10 |
| Tetra | **4091** | 10 |  |  | **3969** | 7.9 |  |  | **9031** | 9 |  |  | **4169** | 7.3 |  |  | **4313** | 5.2 |  |
| AAAT | 2053 | 5 | 12 |  | 2019 | 4 | 10 |  | 4595 | 4.6 | 15 |  | 2002 | 3.5 | 13 |  | 2155 | 2.6 | 17 |
| ACAT | 683 | 1.7 | 348 |  | 558 | 1.1 | 187 |  | 1453 | 1.4 | 348 |  | 700 | 1.2 | 66 |  | 622 | 0.7 | 3155 |
| AATT | 432 | 1 | 10 |  | 391 | 0.8 | 12 |  | 885 | 0.9 | 12 |  | 450 | 0.8 | 11 |  | 456 | 0.5 | 26 |
| AAAG | 391 | 1 | 14 |  | 509 | 1 | 11 |  | 1007 | 1 | 14 |  | 449 | 0.8 | 14 |  | 510 | 0.6 | 38 |
| AATG | 115 | 0.3 | 12 |  | 132 | 0.3 | 10 |  | 259 | 0.3 | 12 |  | 126 | 0.2 | 8 |  | 153 | 0.2 | 10 |
| AAAC | 106 | 0.3 | 13 |  | 109 | 0.2 | 9 |  | 227 | 0.2 | 13 |  | 116 | 0.2 | 11 |  | 105 | 0.1 | 9 |
| AGAT | 57 | 0.1 | 151 |  | 52 | 0.1 | 21 |  | 113 | 0.1 | 151 |  | 69 | 0.1 | 20 |  | 68 | 0.1 | 22 |
| others | 254 | 0.6 | 13 |  | 199 | 0.4 |  |  | 492 | 0.5 |  |  | 257 | 0.5 |  |  | 244 | 0.3 | 14 |
| Penta | **6148** | 14.9 |  |  | **7714** | 15.3 |  |  | **14970** | 14.9 |  |  | **6895** | 12.1 |  |  | **8533** | 10.3 |  |
| AAAAT | 1934 | 4.7 | 14 |  | 3235 | 6.4 | 11 |  | 5556 | 5.5 | 14 |  | 1964 | 3.4 | 14 |  | 3671 | 4.4 | 20 |
| AAAAG | 661 | 1.6 | 9 |  | 802 | 1.6 | 13 |  | 1582 | 1.6 | 13 |  | 654 | 1.1 | 12 |  | 856 | 1.0 | 13 |
| AATCG | 635 | 1.5 | 10 |  | 157 | 0.3 | 8 |  | 850 | 0.8 | 10 |  | 1029 | 1.8 | 9 |  | 249 | 0.3 | 7 |
| AAATT | 595 | 1.4 | 8 |  | 755 | 1.5 | 10 |  | 1450 | 1.4 | 10 |  | 590 | 1 | 9 |  | 828 | 1.0 | 9 |
| AATAT | 582 | 1.4 | 52 |  | 875 | 1.7 | 73 |  | 1641 | 1.6 | 73 |  | 636 | 1.1 | 24 |  | 934 | 1.1 | 36 |
| CCCGG | 199 | 0.5 | 6 |  | 88 | 0.2 | 6 |  | 303 | 0.3 | 6 |  | 362 | 0.6 | 6 |  | 146 | 0.2 | 5 |
| AAAAC | 155 | 0.4 | 8 |  | 171 | 0.3 | 6 |  | 350 | 0.3 | 8 |  | 174 | 0.3 | 6 |  | 200 | 0.2 | 8 |
| AATTC | 133 | 0.3 | 7 |  | 166 | 0.3 | 9 |  | 312 | 0.3 | 9 |  | 125 | 0.2 | 6 |  | 185 | 0.2 | 14 |
| ATATC | 113 | 0.3 | 51 |  | 125 | 0.2 | 113 |  | 251 | 0.3 | 113 |  | 115 | 0.2 | 33 |  | 140 | 0.2 | 19 |
| ACAGG | 1 | 0 | 4 |  | 283 | 0.6 | 11 |  | 317 | 0.3 | 11 |  | 0 | 0 | - |  | 224 | 0.3 | 6 |
| others | 1140 | 2.8 | 18 |  | 1057 | 2.1 |  |  | 2358 | 2.4 |  |  | 1246 | 2.2 |  |  | 1100 | 1.3 | 18 |
| Hexa | **15716** | 38.2 |  |  | **20636** | 40.9 |  |  | **39506** | 39.4 |  |  | **15953** | 28 |  |  | **22223** | 26.7 |  |
| AAAAAT | 3898 | 9.5 | 47 |  | 5261 | 10.4 | 12 |  | 9959 | 9.9 | 47 |  | 3859 | 6.8 | 13 |  | 5794 | 7.0 | 18 |
| AAAAAG | 1676 | 4.1 | 9 |  | 1988 | 3.9 | 12 |  | 4045 | 4 | 12 |  | 1773 | 3.1 | 11 |  | 2263 | 2.7 | 10 |
| AAAATT | 1076 | 2.6 | 13 |  | 1201 | 2.4 | 8 |  | 2434 | 2.4 | 13 |  | 1035 | 1.8 | 9 |  | 1322 | 1.6 | 8 |
| AAAATG | 636 | 1.5 | 10 |  | 489 | 1 | 7 |  | 1181 | 1.2 | 10 |  | 633 | 1.1 | 14 |  | 540 | 0.6 | 10 |
| AAATAT | 594 | 1.5 | 13 |  | 622 | 1.2 | 59 |  | 1314 | 1.3 | 59 |  | 641 | 1.1 | 20 |  | 670 | 0.8 | 12 |
| AATTAT | 424 | 1 | 7 |  | 389 | 0.8 | 8 |  | 890 | 0.9 | 8 |  | 403 | 0.7 | 11 |  | 389 | 0.5 | 9 |
| AAAAAC | 375 | 0.9 | 7 |  | 372 | 0.7 | 13 |  | 801 | 0.8 | 13 |  | 440 | 0.8 | 24 |  | 371 | 0.4 | 6 |
| AAATTT | 366 | 0.9 | 5 |  | 383 | 0.8 | 5 |  | 820 | 0.8 | 5 |  | 425 | 0.7 | 6 |  | 432 | 0.5 | 6 |
| AAAATC | 332 | 0.8 | 14 |  | 246 | 0.5 | 11 |  | 626 | 0.6 | 14 |  | 335 | 0.6 | 26 |  | 224 | 0.3 | 13 |
| AACCCT | 332 | 0.8 | 7 |  | 61 | 0.1 | 6 |  | 421 | 0.4 | 7 |  | 268 | 0.5 | 6 |  | 73 | 0.1 | 7 |
| ACATAT | 316 | 0.8 | 36 |  | 365 | 0.7 | 28 |  | 755 | 0.8 | 40 |  | 341 | 0.6 | 15 |  | 404 | 0.5 | 64 |
| AAAACT | 296 | 0.7 | 45 |  | 151 | 0.3 | 7 |  | 522 | 0.5 | 45 |  | 325 | 0.6 | 14 |  | 116 | 0.1 | 5 |
| AATATT | 204 | 0.5 | 7 |  | 209 | 0.4 | 7 |  | 455 | 0.5 | 7 |  | 243 | 0.4 | 9 |  | 232 | 0.3 | 8 |
| AAATGG | 191 | 0.5 | 11 |  | 216 | 0.4 | 7 |  | 433 | 0.4 | 11 |  | 171 | 0.3 | 8 |  | 211 | 0.3 | 9 |
| AAATTG | 165 | 0.4 | 6 |  | 169 | 0.3 | 5 |  | 353 | 0.4 | 6 |  | 179 | 0.3 | 7 |  | 179 | 0.2 | 6 |
| AACAGT | 117 | 0.3 | 5 |  | 822 | 1.6 | 8 |  | 1007 | 1 | 8 |  | 246 | 0.4 | 5 |  | 883 | 1.1 | 11 |
| AATCAG | 42 | 0.1 | 7 |  | 2590 | 5.1 | 11 |  | 2927 | 2.9 | 11 |  | 43 | 0.1 | 9 |  | 2646 | 3.2 | 41 |
| others | 4676 | 11.4 | 21 |  | 5102 | 10.1 |  |  | 10563 | 10.5 |  |  | 4593 | 8.1 |  |  | 5474 | 6.6 | 190 |
| Total | **41119** |  |  |  | **50443** |  |  |  | **100290** |  |  |  | **56937** |  |  |  | **83160** |  |  |

A_t_, D_t_: two subgenomes of allotetraploid cotton *G. hirsutum*

**Table S3:** Distribution of different motif types in *Gossypium* species (**Separate Table**).

**Table S4:** Pearson correlation between *Gossypium* species.

|  | **A_t_** | **D_t_** | ***G. hirsutum*** | ***G. arboreum*** | ***G. raimondii*** |
| --- | --- | --- | --- | --- | --- |
| **Motif length** | |  |  |  |  |
| A_t_ | 1.0000 | 0.9946 | 0.9984 | 0.5411 | 0.6559 |
| D_t_ |  | 1.0000 | 0.9988 | 0.5508 | 0.6763 |
| *G. hirsutum* | |  | 1.0000 | 0.5454 | 0.6655 |
| *G. arboreum* | |  |  | 1.0000 | 0.9770 |
| *G. raimondii* | |  |  |  | 1.0000 |
|  |  |  |  |  |  |
| **Motif type** | |  |  |  |  |
| A_t_ | 1.0000 | 0.9582 | 0.9915 | 0.6310 | 0.7285 |
| D_t_ |  | 1.0000 | 0.9869 | 0.5908 | 0.7459 |
| *G. hirsutum* | |  | 1.0000 | 0.6202 | 0.7440 |
| *G. arboreum* | |  |  | 1.0000 | 0.9627 |
| *G. raimondii* | |  |  |  | 1.0000 |
|  |  |  |  |  |  |
| **Motif repeat number** | |  |  |  |  |
| A_t_ | 1.0000 | 0.9982 | 0.9995 | 0.9426 | 0.9789 |
| D_t_ |  | 1.0000 | 0.9995 | 0.9434 | 0.9804 |
| *G. hirsutum* | |  | 1.0000 | 0.9433 | 0.9797 |
| *G. arboreum* | |  |  | 1.0000 | 0.9881 |
| *G. raimondii* | |  |  |  | 1.0000 |

A_t_, D_t_: two subgenomes of allotetraploid cotton *G. hirsutum*

**Table S5: The correlation between microsatellites and genes or TEs.**

| Chromosome | Microsatellites | | | |  | Genes | |  | TEs | |
| --- | --- | --- | --- | --- | --- | --- | --- | --- | --- | --- |
|  | Frequency | P x^2^test | r (gene) | r (TE) |  | Frequency | P x^2^test |  | Frequency | P x^2^test |
| A1 | 33 | 6.9E-134 | 0.78 | -0.38 |  | 20 | 0.0E+00 |  | 1418 | 0.0E+00 |
| A2 | 35 | 4.6E-147 | 0.84 | -0.38 |  | 21 | 0.0E+00 |  | 1481 | 0.0E+00 |
| A3 | 34 | 8.1E-127 | 0.76 | -0.14 |  | 19 | 0.0E+00 |  | 1415 | 0.0E+00 |
| A4 | 34 | 2.6E-86 | 0.82 | 0.00 |  | 20 | 1.2E-187 |  | 1421 | 8.9E-196 |
| A5 | 46 | 6.6E-127 | 0.71 | -0.49 |  | 39 | 0.0E+00 |  | 1267 | 0.0E+00 |
| A6 | 31 | 9.8E-148 | 0.78 | -0.12 |  | 18 | 0.0E+00 |  | 1436 | 0.0E+00 |
| A7 | 42 | 2.0E-94 | 0.80 | -0.17 |  | 28 | 3.1E-265 |  | 1375 | 0.0E+00 |
| A8 | 34 | 6.0E-186 | 0.83 | -0.36 |  | 22 | 0.0E+00 |  | 1426 | 0.0E+00 |
| A9 | 41 | 3.7E-129 | 0.76 | -0.42 |  | 29 | 0.0E+00 |  | 1364 | 0.0E+00 |
| A10 | 37 | 1.0E-120 | 0.65 | -0.25 |  | 22 | 0.0E+00 |  | 1404 | 0.0E+00 |
| A11 | 41 | 2.9E-99 | 0.76 | -0.38 |  | 32 | 0.0E+00 |  | 1327 | 0.0E+00 |
| A12 | 40 | 3.7E-162 | 0.84 | -0.38 |  | 29 | 0.0E+00 |  | 1367 | 0.0E+00 |
| A13 | 39 | 3.0E-131 | 0.80 | -0.19 |  | 26 | 0.0E+00 |  | 1425 | 1.4E-294 |
| **Total A_t_** | **37** | **2.0E-87** | **0.78** | **-0.28** |  | **25** | **9.3E-189** |  | **1394** | **6.8E-197** |
| D1 | 49 | 3.0E-57 | 0.78 | -0.12 |  | 37 | 7.9E-216 |  | 1231 | 4.4E-218 |
| D2 | 46 | 2.0E-46 | 0.75 | -0.07 |  | 35 | 9.5E-285 |  | 1266 | 1.1E-215 |
| D3 | 48 | 7.2E-56 | 0.82 | 0.52 |  | 36 | 4.5E-141 |  | 1197 | 3.3E-142 |
| D4 | 49 | 3.3E-33 | 0.80 | 0.11 |  | 37 | 7.0E-128 |  | 1280 | 2.7E-80 |
| D5 | 57 | 7.8E-27 | 0.63 | -0.43 |  | 60 | 4.4E-264 |  | 1029 | 0.0E+00 |
| D6 | 46 | 2.2E-33 | 0.75 | 0.13 |  | 35 | 7.8E-195 |  | 1255 | 1.8E-139 |
| D7 | 54 | 4.1E-32 | 0.78 | 0.01 |  | 43 | 9.2E-122 |  | 1214 | 5.6E-190 |
| D8 | 49 | 9.6E-61 | 0.82 | -0.09 |  | 40 | 2.0E-223 |  | 1242 | 2.0E-214 |
| D9 | 52 | 1.5E-33 | 0.68 | -0.34 |  | 47 | 1.0E-221 |  | 1159 | 0.0E+00 |
| D10 | 48 | 1.0E-26 | 0.59 | 0.02 |  | 38 | 2.1E-189 |  | 1216 | 3.6E-231 |
| D11 | 51 | 8.9E-29 | 0.66 | -0.27 |  | 50 | 2.1E-272 |  | 1155 | 0.0E+00 |
| D12 | 51 | 1.4E-37 | 0.78 | -0.20 |  | 45 | 1.8E-214 |  | 1179 | 2.6E-239 |
| D13 | 50 | 5.2E-54 | 0.82 | -0.04 |  | 41 | 1.2E-198 |  | 1227 | 9.4E-192 |
| **Total D_t_** | **50** | **1.4E-27** | **0.74** | **-0.06** |  | **42** | **7.1E-123** |  | **1204** | **2.1E-81** |

A_t_, D_t_: two subgenomes of allotetraploid cotton *G. hirsutum*

**Table S6:** Summary of all 77,996 microsatellite markers (**Separate Table**).

**Table S7:** Evaluation of amplification and amplified fragment of the selected microsatellite markers (**Separate Table**).

**Table S8:** Distribution of polymorphic microsatellite markers between TM-1 and Hai7124.

| **Chromosome** | **Number of polymorphic marker** | **Chromosome** | **Number of polymorphic marker** |
| --- | --- | --- | --- |
| A01 | 244 | D01 | 330 |
| A02 | 276 | D02 | 463 |
| A03 | 211 | D03 | 332 |
| A04 | 229 | D04 | 203 |
| A05 | 360 | D05 | 362 |
| A06 | 394 | D06 | 416 |
| A07 | 412 | D07 | 381 |
| A08 | 345 | D08 | 333 |
| A09 | 270 | D09 | 313 |
| A10 | 359 | D10 | 354 |
| A11 | 390 | D11 | 371 |
| A12 | 502 | D12 | 278 |
| A13 | 454 | D13 | 243 |
| **A_t_** | 4,446 | **D_t_** | 4,379 |
| **All** | **8,825** |  |  |

A_t_, D_t_: two subgenomes of allotetraploid cotton *G. hirsutum*
